# Busting the Baby Teeth Myth and Increasing Children’s Consumption of Tap Water: Building Public Will for Children’s Oral Health in Colorado

**DOI:** 10.3389/fpubh.2017.00238

**Published:** 2017-09-11

**Authors:** Wyatt C. Hornsby, William Bailey, Patricia A. Braun, Karl Weiss, James Heichelbech

**Affiliations:** ^1^Delta Dental of Colorado Foundation, Denver, CO, United States; ^2^School of Dental Medicine, University of Colorado, Aurora, CO, United States; ^3^School of Medicine, University of Colorado Anschutz School of Medicine, Denver, CO, United States; ^4^HealthCare Research, Inc., Denver, CO, United States

**Keywords:** oral health, behavior change intervention, sugary drinks, fruit juice, early childhood caries

## Abstract

**Question:**

Can a multifaceted statewide communications campaign motivate behavior change in low-income Colorado families to limit children’s fruit juice consumption and increase children’s consumption of tap water to prevent tooth decay?

**Purpose:**

Caries is the most common chronic disease of childhood, affecting 40% of kindergartners and 55% of third graders in Colorado. Frequent consumption of 100% fruit juice is linked to childhood caries. The purpose of this campaign, “Cavities Get Around,” was to motivate families to limit children’s fruit juice consumption and increase consumption of tap water to protect baby teeth from caries, while also building public will for children’s oral health.

**Methods:**

The campaign included targeted media, promotores/organizers, and family education. We focused on fruit juice because audience research showed many families view fruit juice as healthy, and it is also a common beverage among young children up to age of 6 years. We also focused on low-socioeconomic status families because data show higher childhood tooth decay rates in this population. To evaluate progress, we conducted identical pre- and post-surveys, each of 600 random low-income parents contacted by landline, mobile telephone, and Internet, allowing for comparative data.

**Results:**

Significant progress was achieved compared to 2014 baseline results. Findings from a November 2015 statewide survey of parents included the following: (1) 22-point increase from 2014 in percentage of children regularly drinking tap water (from 41 to 63%). (2) 29-point decrease from 2014 in percentage of respondents who considered fruit juice consumption important to their child’s health and nutritional needs (from 72 to 43%). (3) 19-point reduction in fruit juice consumption among young children (from 66% in 2014 to 47% in 2015). (4) 6-point reduction in percentage of parents considering baby teeth “less important” than adult teeth (from 21% in 2014 to 15% in 2015). The campaign also played a role in new state rules prohibiting childcare centers from serving sugar-sweetened beverages and capping 100% juice to twice per week.

**Conclusion:**

The campaign development, strategies, and evaluation results are instructive for others working on health promotion, childhood nutrition, and education interventions.

## Introduction

Dental caries, or tooth decay, is the most common chronic disease of childhood, with rates of disease at epidemic proportions, and yet it remains a “silent epidemic.” (1) Early childhood caries is characterized by one or more decayed, missing, or filled teeth in children under the age of 6 years. The disease is transmissible and caused by multiple factors (2). Bacteria, *Streptococcus mutans*, and others produce strong acids when they metabolize sugars from foods and beverages. The acids erode tooth enamel, resulting in a cavity. Transmission of cavity-causing bacteria from a caregiver to child can occur. Caries has been termed a “diet-mediated disease” because sugars fuel the disease process (3). Consuming fruit juice^1^ and sugar-containing beverages and snacks, especially between meals and before bed, elevates the risk of caries in children (4).

Untreated caries can be painful. For children, this can mean difficulty concentrating, speaking, and eating. A 2000 U.S. Surgeon General report, “Oral Health in America,” found that more than 51 million school hours are lost every year due to dental-related illness (1). Untreated caries in young children can lead to premature loss of primary, or “baby,” teeth, and disease in primary teeth can “spread” to erupting adult, or permanent, teeth. Children with oral pain earn lower grade point averages than children without oral pain (5). Sometimes, the treatment provided for extensive caries in children’s teeth is visible stainless steel crowns, or “silver caps.” Children with extensive tooth decay may require treatment under general anesthesia.

In Colorado, as in most other states, dental caries in young children is a serious public health concern with substantial human and financial costs. A 2012 report by the Colorado Department of Public Health and Environment (CDPHE) indicated that 39.7% of kindergarten children and 55.2% of third graders experienced caries, or tooth decay. Nearly one in seven children in both age groups (13.8% of kindergartners and 14.4% of third graders) experienced untreated tooth decay. In addition, the report revealed ethnic disparities. Among kindergarteners, 55.0% of Hispanic children and 38.0% of black children experienced caries compared to 31.9% of white children. Among children in third grade, 69.5% of Hispanic children and 56.4% of black children compared to 48.1% of white children experienced caries. Among children in kindergarten, untreated decay was higher among Hispanic children (18.5%) compared with black (16.8%) and white (11.4%) children (6). Income disparities also exist. The CDPHE report found that, in schools with at least 75% of students enrolled in free and reduced lunch programs, caries experience was as high as 53.1% in kindergartners and 73.4% in third graders (6).

Establishing good oral health in children can be a daunting and complex challenge, especially in low-income families (7). Access to care is one challenge. In 2015, according to the Colorado Health Access Survey, only 11.8% of children enrolled in Medicaid and the State Children’s Health Insurance Program (SCHIP) had a dental checkup by age of 1 year, a recommendation for all children (8). The survey also indicated that, among Medicaid- and SCHIP-enrolled children up to 6 years of age, only one out of three had a dental exam in the previous 12 months (8). Thirty-seven percent of children aged 0–6 years from families earning less than 200% of the federal poverty level had not visited a dentist in the previous 12 months (8). Many dental providers are unwilling to see young children, especially children enrolled in public insurance programs. A 2015 report by the Colorado Health Institute reported that only 877 of the 2,654 practicing dentists in Colorado had provided services to at least one Medicaid enrollee in 2013–2014 (9).

Oral health behaviors and attitudes are another challenge. For example, many dental professionals working with pediatric patients report a common belief, or myth, among parents that baby teeth are of limited value since they “fall out anyway.” This perception of baby teeth translates into less urgency to care for them. Poor nutrition (10), particularly overconsumption of sugars along with inadequate exposure to fluoride, is often another challenge. Fluoride helps prevent cavities. In Colorado, 74.91% of residents served by public water systems have access to optimally fluoridated water (11). Yet, among some immigrant populations, there is often skepticism about the safety of consuming water from public supplies (12), leading many children to consume fruit juices and other sugary drinks instead of tap water.

Putting these challenges together—insufficient access to oral health care, limited consumption of fluoridated tap water, high consumption of fruit juices and other sugar-containing beverages and snacks, and the belief that baby teeth have little value—it is not surprising that tooth decay rates are high among some Colorado families, especially those lower on the socioeconomic spectrum. Yet these challenges are not insurmountable. Recognizing that oral disease is largely preventable, Colorado’s governor, in 2011, named oral health one of the state’s “10 Winnable Battles,” positioning the issue as a public concern (13).

Delta Dental of Colorado Foundation (DDCOF), guided by its mission to eradicate childhood tooth decay, launched a statewide initiative, using a “public will building” model, to establish children’s oral health as a priority among community decision-makers and “influencers,” policymakers, and families living at or below 200% of the Federal Poverty Level with children aged 0–6 years (14). The 0–6 years of age range was chosen because DDCOF’s mission focuses on eradication of tooth decay in children.

Driving DDCOF’s public will building initiative was the hypothesis that, if children’s oral health were to become a priority for families, influencers, and policymakers, tooth decay could be prevented. Reducing children’s access to and consumption of fruit juice, while increasing consumption of tap water, to protect baby teeth from decay became the focal point of this work from 2014 to present. This report details the development, strategies, and evaluation results associated with DDCOF’s public will building program, specifically the “Cavities Get Around” campaign, as a potential model for others working on health promotion, childhood nutrition, and education interventions, and provides insights for additional child health initiatives.

## Materials and Methods

### Overview of Public Will Building Development

The audience research that was conducted to inform development of the initiative was done within the context of a public-facing campaign with social marketing elements. The campaign was conducted over two large phases, the first from 2011 to 2013 and the second starting in 2014 (each will be described below). The results of the first phase informed the development and implementation of the second phase, which is ongoing.

(1) The primary objective of the Phase I work (2011–2013) was to better understand the problem of poor children’s oral health, develop and launch a pilot public will building campaign focused on parents and their children brushing together, and assess effectiveness. Research informing this phase included in-home visits and focus groups with low-income families, interviews with experts and influencers, literature reviews, and an environmental scan. We used the findings from these activities to develop messaging that informed development of television and radio advertisements, a promotores de salud program (health promoters), and community partnerships. To evaluate the impact of this work, we conducted pre- and post-surveys of randomly selected low-income families.
(2) We adopted a different approach in Phase II (2014–present) based on findings from additional research involving low-income families and experts. Our Phase II primary objectives were to prevent tooth decay in children’s baby teeth by increasing children’s access to and consumption of tap water and decreasing their access to and consumption of fruit juice and other sugary drinks. We conducted focus groups, a statewide survey, and interviews and used the findings to develop a community-based campaign. The impact of the work was, and continues to be, measured from statewide survey results, which will be shared here.

#### Phase I (2011–2013)

Delta Dental of Colorado Foundation wished to use public will building to not only modify oral health behaviors but also to change social norms, systems, and policies at the institutional and public levels—all “from the ground up.” This was the approach many advocates in the childhood obesity movement were using (15). Planning began in 2011, when DDCOF hired The Metropolitan Group, a Portland, Oregon firm with expertise in public will building. Pre-campaign research was conducted in Colorado in 2011 and 2012 (Table 1).

**Table 1:** Public will building for children’s oral health in Colorado, Phase I pre-campaign research activities (2011–2012).

Activity	Type	Purpose
Background data scan	Interviews of children’s oral health experts	Identified segments of population most affected by tooth decay
	Conducted literature review	

Qualitative research with low-income families	Visits to homes	Through visits with 49 families, assessed perspectives of populations most affected by early childhood caries
	Visits in clinics	

Qualitative research with low-income pregnant women and parents of young children	Focus groups (2)	Shared preliminary messages

Qualitative research with influencers	Focus groups (2)	Identified attitudes, knowledge, values, and motivators that might increase prioritization of children’s oral health

Quantitative research	Random telephone survey	Established baselines for measuring progress over time

Brush with me campaign (launched April 2013)	Messaging campaign	Messages focused on behavior change, including better home care and parents and children brushing their teeth together. There was a secondary message of putting only water in sippy cups between meals and at bedtime

Post-assessment survey of brush with me campaign	Online survey	Assessed effectiveness of messaging campaign with low-income families in Denver metro area

Following multiple research activities and analyses, the bilingual “Brush with Me” pilot campaign launched in April 2013. Focusing on behavior change within low-income populations in greater Denver, the pilot campaign led with the message, “Begin brushing your children’s teeth, and your teeth, together every day.” A secondary message of, “Between meals and at bedtime, put only water in your child’s sippy cup or bottle,” was limited only to the campaign website. The brushing message was conveyed through paid advertising and mass media, promotores de salud (health promoters within the Hispanic community), public events, and education programs with partners such as Children’s Museum of Denver at Marsico Campus—all focusing on the Denver area.

The television advertisement ran on both English- and Spanish-speaking channels for much of the last 8 months of 2013. It showed a young girl with severe decay in an operating room and implored parents to brush their teeth with their children. This creative direction was tested in focus groups with low-income parents. Parents were told how inadequate child oral health could result in the need for emergency care requiring general anesthesia and how brushing their teeth with their child could help prevent cavities and create new healthy traditions. Avoiding the pain of untreated cavities resonated with them; many had experienced that pain firsthand and did not want it for their child. We shared that visits to emergency departments due to oral health problems in children were far more common than many realized. We showed how poor oral health could affect not just children but also their community. Participants told us this approach would get the attention of the communities they represented.

In late 2013, a post-assessment online survey of “Brush with Me,” conducted by the campaign with 203 low-income families living in the Denver metro area, revealed no significant progress compared to a baseline telephone survey from February of the same year. This could have been due to the two surveys using different methodologies, underscoring a suboptimal evaluation framework. We decided to conduct additional research with low-income families and oral health experts to verify if the “Brush with Me” message was effective and, if not effective, identify a new direction. During this assessment, advertising of the “Brush with Me” message ceased.

#### Early Phase II (2014–2015)

The next phase of research began in early 2014, when a Denver, Colorado-based firm, HealthCare Research, Inc., was hired. The firm began the additional research to better understand the perspectives of the target population (low-income families with children ages 0–6 years) we wished to reach, gain new insights into the problem we were trying to address, and determine if “Brush with Me” provided an effective campaign direction. These research activities included qualitative and quantitative elements (Table 2).

**Table 2:** Public will building for children’s oral health in Colorado, Phase II pre-campaign research activities (2014).

Activity	Type	Purpose
Qualitative research with oral health experts	Interviews of pediatric and general dentists, hygienists, and oral health professionals	To gain better understanding of the challenges providers experienced and identify solutions they believed could make the biggest difference in improved oral health

Exploratory qualitative research with low-income parents	Parent focus groups, English and Spanish (4)	To explore attitudes and behaviors toward unnecessary pain and suffering from tooth decay, the possibility of being cavity-free for life, the toll of painful cavities on school performance and attendance, the importance of aesthetics, the lifelong impact of poor oral health, and creating positive oral health behaviors at a young age

Baby teeth matter message platform and concept testing	Campaign development; creative concept testing	To synthesize the findings from the expert interviews and exploratory parent focus groups, and then outline desired message tone, why baby teeth matter, how cavities form, what parents/caregivers can do, and the benefits of desired behaviors

Creative concept testing qualitative research with low-income parents	Parent focus groups, English and Spanish (4)	To test potential creative directions for a new campaign. Results from the testing were obtained through a four-step process and informed the direction that was chosen

Pre-campaign statewide survey of low-income families (2014)	Telephone and online survey of 603 families	To assess reactions to message content derived from the expert interviews and exploratory focus groups, and establish a baseline of results to measure future progress related to the perceived importance of baby teeth and attitudes and behaviors related to children’s fruit juice and water consumption. Coding techniques were used to synthesize responses to the open-ended questions

In addition, we conducted behavior change modeling exercises to help us identify, as the research ensued, where low-income families might be in their attitudes, awareness, and actions related to children’s oral health. The model we used, based on the Stages of Change from the Transtheoretical Model, showed the common steps in the behavior change process: (1) becoming aware of the issue, (2) forming a personal connection to the issue, or experiencing an “awakening,” (3) contemplating making a change, (4) taking small steps toward change, and (5) committing to the change. We also developed a new logic model and evaluation framework (Table 3).

**Table 3:** Delta Dental of Colorado Foundation public will building logic model (developed in 2014).

Inputs: What resources are needed? Funding Reliable quantitative and qualitative data Evaluation framework to gauge success Partners and synergies	Activities: What needs to be done to create early momentum? Targeted advertising Public relations activities Family engagement and promotores Community organizing Policy advocacy Network building through partnerships	Outputs: How do we know we are completing important work? Number of impressions Number of contacts Number of participants Connections among decision-makers and leaders

Short-term outcomes: What do we need to achieve in years 1–3?	Intermediate outcomes: What is the path to success?	Long-term outcomes: What are we trying to accomplish?

Increased awareness among low-income families that: Baby teeth matter because cavities can spread from baby teeth to adult teeth The sugar content in fruit juice is harmful to baby teeth, and so juice should be limited to mealtimes Tap water is healthy and is what children should drink between meals and at bedtime	Children’s oral health is prioritized such that: Social norms change (priorities and expectations) Commitment happens at the family and community levels Policies change at the institutional and public levels to support healthy baby teeth	Eradication of cavities among children in Colorado

### Strongest Campaign Concept

From analyses of focus group and quantitative survey findings, we determined that the most effective campaign concept was “Cavities Get Around.” The concept showed how “cavities can spread from baby teeth to adult teeth” and implored, “Don’t let that happen to your child’s mouth.” Focusing on the importance of baby teeth, limiting fruit juice consumption, and serving more water, “Cavities Get Around” was flexible enough to allow for a behavior change effort as well as the larger goals of building public will for children’s oral health. “Cavities Get Around” was especially appealing since the idea of cavities spreading from baby teeth to adult teeth was new to and memorable with the population we sought to reach.

### Phase II Campaign Goals

The “Cavities Get Around” campaign launched in August 2014. To map out how the campaign would evolve over time, we created a logic model describing short-term, intermediate, and long-term outcomes of the work (Table 3).

- Short-term: through targeted advertising, social media, promotores de salud, educational programs, text messaging, and other means, raise awareness of the importance of baby teeth, the oral health impacts of unlimited fruit juice consumption, and the benefits of water, especially fluoridated tap water, on children’s oral health. Also, achieve behavior change, specifically more families serving children only tap water between meals and at bedtime.
- Intermediate: create new positive social norms for children’s oral health through local community engagement, using trusted ambassadors such as promotores de salud, organizers, and influencers. If community residents, committed to the “movement,” joined together, could they create new norms around children’s oral health such that cavity-free children were the expectation? By the same token, could communities change local practices to create healthier environments, with water and not fruit juice and other sugary drinks as the default beverage for children? Further upstream, could momentum in communities, bolstered by public relations activities, then spark public policy interventions, such as measures to curb consumption of sugary drinks, expand access to water, improve access to oral health care, etc.?
- Long-term: if we could generate momentum in communities, we could feasibly create a Colorado where eradication of cavities may become realistic.

### Advertisements

Advertising was among the multiple components of the campaign. Reflecting our research learnings, a new advertisement, released in English and Spanish, had a light tone to it, while still delivering an important, actionable message. Instead of adding to the stress and “noise” already encountered by the families we wished to reach, we chose to provide quality health information in a fun and approachable manner. This was embodied in our “sugar troll” advertisement, the first commercial released under the new “Cavities Get Around” campaign.^2^

The 30-s advertisement depicted a mother and her young child sitting at a kitchen table in a modest home. The mother is about to pour her child a glass of fruit juice when a “sugar troll” appears. The Muppet-like creature, meant to represent poor oral health, has decayed teeth and is visibly happy that the mother is about to serve the fruit juice to her child. In the midst of his excitement as the mother is reaching for the juice, he loses a tooth, which falls to the floor. Suddenly, the mother has second thoughts and puts the juice back down on the table and reaches for a pitcher of tap water. Meanwhile, the sugar troll is visibly upset by her healthier choice and then retreats. The child drinks the water. The voice-over in the advertisement has meanwhile been talking in simple terms—and tracking with the scene—about the importance of baby teeth, how cavities can spread, how the sugar in juice can be harmful to children’s oral health, and why water is beneficial. A “Sugar Troll” radio advertisement (English and Spanish) was also developed.

In 2015, a second, complementary set of visual advertisements was developed. In these English and Spanish advertisements, various junk foods, such as donuts and churros, were blended and then the contents placed next to a fruit juice container to show equal amounts of sugar in both items.

Advertising included television (English and Spanish outlets), traditional radio, digital/online (including online radio), billboards, and social media. The advertising and creative agency, Amélie Company in Denver, Colorado, targeted all paid media at low-income mothers ages 18–34 across Colorado.

### Partner Assistance

In addition to advertising, various outreach programs were implemented through partners. Curricula for training promotores de salud were developed for two partners (see below). All partners agreed to be tightly coordinated in delivering the core messages related to the importance of baby teeth, limiting fruit juice to mealtimes, and serving only water, particularly tap water, between meals and at bedtime. To maximize impact, each partner was provided with various bilingual giveaways for families, including brochures about why baby teeth matter and why water, not fruit juice, is the healthiest beverage to serve to young children; sippy cups that said “only water, please” in English and Spanish; and posters, magnets, and other materials. Partners included:

- Children’s Museum of Denver at Marsico Campus, an influential non-profit organization, launched three “Healthy Smiles” oral health programs. The programs included a weekly story time, the no-cost “Molar Expedition” program for Title I schoolchildren, and a summer in-museum offering for families that has since been replaced with a live “Sugar Scaler” demonstration for families in the museum’s teaching kitchen.
- A promotores de salud (Hispanic, underserved and rural community health educators) and, eventually, coalition building program launched in Pueblo through a partnership with Southeastern Colorado Area Health Education Center. A curriculum for training the promotores was developed in 2014 and fully implemented in 2015. The curriculum aligned with “Cavities Get Around” messaging.
- A grassroots community organizing initiative through Westwood Unidos launched in Southwest Denver, an area disproportionately affected by childhood obesity and heavily populated by Latino families (16). A “Cavities Get Around” curriculum for training the organizers, similar to the Southeastern Colorado Area Health Education Center curriculum, was developed in 2015.
- Bright By Three (then known as Bright Beginnings) implemented “Cavities Get Around”-specific oral health messaging in its home visitation programs for families with children infant to 3 years of age. Bright By Three also added oral health messages to a statewide text messaging campaign it was launching.
- Qualistar Colorado began training child care providers in Cavity Free Kids, an existing oral health education curriculum for children with messages aligning with the “Cavities Get Around” campaign. Cavity Free Kids teaches five basics of children’s oral health: the importance of baby teeth; drinking water for thirst; eating tooth-healthy foods; brushing, flossing and swishing; and going to a dental provider.
- DDCOF’s Colorado Medical-Dental Integration (CO MDI) Project, a program integrating registered dental hygienists in medical office settings to improve access to care for low-income populations. “Cavities Get Around” materials, such as bilingual brochures, magnets, and posters, were distributed to CO MDI hygienists and clinics.

### Media Outreach

We increased efforts to engage influential media outlets to start covering the issue of children’s oral health. The goal of these earned-media efforts was to drive awareness among influencers and policymakers of the epidemic of poor child oral health and start “planting seeds” for the public will building aspects of the work. Notable examples of key media exposure included a story on Colorado Public Radio (CPR) about the “silent epidemic” of tooth decay in children and another story on CPR about efforts to engage Latino communities on drinking tap water. The second story was later picked up by National Public Radio and ultimately covered in a slightly different way by *The New York Times*. Opinion editorials about policy issues, such as new requirements announced by the Food and Drug Administration for the nutrition facts label and the Colorado healthy beverage policy for schools, also created opportunities to reach influencers. In addition, we leveraged paid media partnerships to create quality editorial coverage, such as the campaign director appearing on popular morning new programs to blend donuts to show how much sugar is in fruit juice. Social media helped expand the reach of this exposure.

### Policy

In addition to the social renorming and behavior change work through the “Cavities Get Around” campaign, we joined efforts to support policy opportunities as they surfaced. This included developing alliances with organizations working to reduce and prevent childhood obesity specifically through efforts to curb children’s consumption of and access to sugary drinks. For example, in 2015, the campaign joined a large coalition effort to support, and advise on, the nutrition components of a set of draft rules the State Board of Human Services was considering for all licensed childcare centers in Colorado. In addition, DDCOF funded a non-profit organization, Healthier Colorado, to support efforts to educate the public about community water fluoridation and its benefits to oral health.

### Phase II Mid-Campaign Statewide Survey of Low-Income Families (2015)

In November 2015, nearly 18 months after the “Cavities Get Around” campaign launched, we conducted a second statewide survey to evaluate progress. Using similar methods as used in the 2014 baseline survey, we surveyed 600 randomly selected low-income parents from across Colorado, who were contacted by landline, by mobile telephone, and online, and were asked many of the same questions, especially those related to beverage consumption, the importance of baby teeth, and attitudes and behaviors related to brushing and visiting a dental provider. The same inclusion criteria used for the 2014 survey were applied to the 2015 survey: (1) must reside in the state of Colorado; (2) must have a child living at home between 6 months of age (provided they have their first baby teeth) and 6 years of age; (3) must be the person in the household who oversees the health care needs of the child; and (4) must have an annual household income below 200% of the 2015 federal poverty guidelines.

### Analytic Approach for 2014 and 2015 Statewide Surveys

The completed interviews for the 2014 and 2015 statewide surveys yielded a maximum margin of sampling error of ±4 percentage points at the 95% level of confidence. Once the data for both surveys were weighted by geography to reflect U.S. Census estimates and checked for accuracy and integrity, the results were tabulated and analyzed using the same approach.

Statistical significance testing was employed when looking at differences between various participant segments/groupings within the data as well as between the 2014 and 2015 statewide assessments included in the results. Only differences that could be cited as being of statistical significance at the 95% level of confidence were reported or if that level of confidence was not achieved.

When comparing differences between various demographic or geographic segments within a single cross-sectional dataset, *t*-tests of means or proportions were used, comparing the percentage (or mean) for that group to everyone else (meaning the group of interest was excluded from the comparison). Testing was performed at the 95% level of confidence using IBM SPSS Statistics for Windows, Version 19.0. Two-tailed hypothesis testing was employed, and variances were tested for homogeneity using Levene’s Test of Equality of Variances.

When testing for differences *between* the two cross-sectional statewide surveys (2014 and 2015), independent *t*-tests of proportions and means were once again used for identifying differences which were of statistical significance at the 95% level of confidence. Two-tailed hypothesis testing was employed (H_o_: *u*_pre_ = *u*_post_), providing a more conservative test result than if the null hypothesis were presented with the assumption that the campaign’s message had created the desired impact on the variables of interest (i.e., H_o_: *u*_pre_ < *u*_post_).

In comparing the results of the 2014 and 2015 statewide surveys, of greatest interest to us was if there had been progress related to the key goals of the campaign:

- Did more parents and caregivers understand that the sugar in fruit juice was harmful to children’s oral health and, as such, reduce how much they were serving their child(ren)?
- Did more parents and caregivers understand the oral health benefits of tap water for children and, as such, increase how much they were serving to their child(ren)?
- Did more parents and caregivers understand the importance of baby teeth, i.e., baby teeth are just as important as adult teeth?

### Ethics Approval Statement

The 49 in-home and in-clinic interviews with families in early Phase I were approved by the Center for Research Strategies in Denver, CO, USA. We completed an application for this work and met the requirements for the center’s ethical evaluation and approval of the study. Phase II did not require Institutional Review Board approval as it was a social campaign. The data presented are from a program evaluation.

## Results

### Expert Interviews

The expert interviews were intended to provide a solid scientific basis for the ensuing research with low-income families. A “storyline” emerged from the key themes from the interviews (Table 4). The storyline “connected the dots” between behaviors and long-term outcomes and brought the realization that any effort to prevent caries in young children had to begin by establishing an understanding of why baby teeth matter. This “value proposition” had to be the central motivation for families and the community to care about preventing early childhood caries to the extent that they would change behaviors, norms, and environments.

**Table 4:** Key themes from expert interviews (conducted in 2014).

Key themes—what the experts told us	Layman’s storyline—what it meant to families with young children
Among many parents and caregivers, there is a lack of understanding of the importance of baby teeth. The thinking is that baby teeth are expendable since children will lose them anyway	Cavities can spread from baby teeth to adult teeth, setting a child up for a lifetime of oral health challenges

Sugary beverages, especially fruit juice served in sippy cups, contribute to poor oral health in children just as much as inadequate teeth brushing	Sugar serves as fuel for bacteria that live in children’s mouths. These bacteria, when fueled by sugar, create acid that eats away at the thin enamel of children’s baby teeth, leading to cavities

Families from Mexico often do not drink tap water because of water safety issues in their native country. This practice in turn reduces exposure to fluoride and increases the consumption of sugary beverages	Tap water is safe and healthy, and the fluoride in tap water protects teeth against decay

### Exploratory Parent Focus Groups

The exploratory focus groups revealed a common perception of baby teeth as disposable and transitory. We heard that almost every parent *said* their child’s baby teeth were important, but they did not know why, and thus there was little motivation to care for them, especially when juxtaposed against all of the other priorities of parenting. While nearly all parents told us their children’s teeth were brushed every day, they were willing to talk about how often they “skipped” brushing, especially if their child had fallen asleep before going to bed or was feeling unwell. Many parents also told us they regularly sent their children to bed with milk or some type of sugary drink to help them sleep. A few commented in the focus groups that they felt overwhelmed by the large amount of milk and fruit juice received though the Women, Infants, and Children’s Program. The information we shared about the importance of baby teeth—for example, cavities could spread from baby teeth to adult teeth and sugar fueled cavity-causing bacteria—was new to almost everyone. This information in turn led several participants to tell us in the focus groups that they desired a change in their behaviors. Many left the sessions saying they would change their oral health practices at home because of what they had just learned.

Connecting the findings from our exploratory focus groups to our previous behavior change modeling exercises, we realized that our public will building work had to start with basic awareness building related to why baby teeth matter and simple ways to protect them from decay.

### Statewide Surveys

The sociodemographic characteristics of the 2014 and 2015 statewide survey cohorts were similar (Table 5). The majority of families lived in the Denver Metro area or Front Range. Slightly less than half of those surveyed were Hispanic.

**Table 5:** Comparison of methodologies between 2014 and 2015 statewide surveys in Colorado.

	April 2014 sample size	November 2015 sample size
**Geographic region**		
Denver Metro	273	279
Front Range	120	125
Southern	90	81
Eastern	48	47
Mountains/West	72	68
Totals	603	600
**Hispanic/Spanish speaking**		
Of Hispanic origin	44%	42%
Spanish speaking	26%	25%

#### 2014 Survey Results

The findings from the 2014 survey of low-income households led to the focus on the importance of protecting baby teeth by serving only water (particularly tap water), and not fruit juice, to children between meals and at bedtime (Table 6).

**Table 6:** Results from 2014 statewide survey^a^ of 603 low-income households.

*Key findings* Although 87% of surveyed parents reported that their children’s teeth were brushed, only 1% of those surveyed reported they brushed their child’s teeth for 2 min, twice a day, every day. Thirteen percent reported their child was too young to brush, which included 34% of those under 2 years of age, 15% of children 2–3 years of age, and 5% of children 4–5 years of age. Thirty-five percent reported they had taken (or planned to take) their child to the dentist by the age of 1 year. Eight-seven percent of parents said their child drank fruit juice at least several times a week, and 55% said the beverage their child was most likely to be walking around with during the day was fruit juice. Seventy-two percent of parents said they believed fruit juice to be important to the health and nutrition of their child.

*Winning messages (after coding responses)* Importance of baby teeth: it is important to take care of your child’s baby teeth because cavities in their baby teeth can easily spread to their adult teeth. How cavities form: sugar is like fuel for cavities. Sugar is found not only in sweets like candy but also in fruit juice, soda, and chocolate milk. What parents can do: parents should limit their children’s consumption of fruit juice and other sugary drinks to mealtimes, and serve only tap water between meals and especially at bedtime. Benefits of child oral care: not only will all of this help prevent cavities but also parents will give their child a beautiful smile; healthy, white teeth; and clean, fresh breath.

*^a^Results include telephone and online survey*.

#### 2015 Survey Results

Thirty-nine percent of all 2015 survey respondents reported they had seen the “Cavities Get Around” campaign advertising; 46% of participants living in Denver reported they had seen the advertising. Comparisons of the November 2015 statewide survey (all respondents) to the 2014 baseline results (Table 7) include the following:

- Increase in the percentage of children aged 0–6 years regularly drinking tap water: 41% (2014), 63% (2015), *p* < 0.01.
- Increase in reported tap water consumption in children aged 2–3 years: 39% (2014), 64% (2015), *p* < 0.01 and children aged 4–6 years: 45% (2014), 69% (2015), *p* < 0.01.
- Increase in reported tap water consumption in Hispanic children: 34% (2014), 60% (2015), *p* < 0.01.
- Decrease in reported perception that fruit juice is important to their child’s health and nutritional needs: 72% (2014), 43% (2015), *p* < 0.01.
- Decrease in reported daily fruit juice consumption among young children aged 0–6 years: 66% (2014), 47% (2015), *p* < 0.01. Broken down further, the reductions in fruit juice consumption were as follows:
  ○ Children aged 6 months to 1 year: 55% (2014), 32% (2015), *p* < 0.01.
  ○ Children aged 2–3 years: 69% (2014), 48% (2015), *p* < 0.01.
  ○ Children aged 4–6 years: 69% (2014), 49% (2015), *p* < 0.01
  ○ All Hispanic children (ages 0–6 years): 68% (2014), 46% (2015), *p* < 0.01.
- Reduction in the percentage of parents who considered baby teeth “less important”: 21% (2014), 15% (2015), *p* < 0.01.

The results also indicated the following:

- Decrease in reported daily consumption of plain (unflavored) milk among children aged 0–6 years: 90% (2014), 82% (2015), *p* < 0.01.
- Increase in reported daily consumption of flavored milks among children aged 0–6 years: 13% (2014), 23% (2015), *p* < 0.01. Additional breakdowns for flavored milk consumption included:
  ○ Reported increase among children aged 2–3 years: 13% (2014), 29% (2015), *p* < 0.01.
  ○ Reported increase among children aged 0–6 years living in the Denver metro area: 11% (2014), 23% (2015), *p* < 0.01.
  ○ Reported increase among children aged 0–6 years living in the Front Range (excluding Denver metro area): 13% (2014), 29% (2015), *p* < 0.01.
  ○ Reported increase among Hispanic children aged 0–6 years: 15% (2014), 29% (2015), *p* < 0.01.

**Table 7:** Comparison of results from first and second statewide surveys of low-income families conducted in April 2014 and November 2015.

	April 2014 (%), *N* = 603	November 2015 (%), *N* = 600	*p*-Value*
**Importance of baby teeth compared to adult teeth**			
Much more important	3	7	<0.01
Somewhat more important	4	7	0.02
Equally important	71	70	n/s
Somewhat less important	16	12	0.05
Much less important	5	3	0.04
**Which of the following types of beverages does your child drink? How often do they drink… (% “daily”)?**			
Tap water	41	63	<0.01
Bottled water	57	56	n/s
Fruit juice	66	47	<0.01
Flavored milk	13	23	<0.01
White milk	90	82	<0.01
Soda	2	3	n/s
**How important is it for your child to drink (item) for their health and nutrition? (% “extremely” and “very important”)**			
White milk	96	88	<0.01
Tap water	43	57	<0.01
Bottled water	65	60	n/s
Fruit juice	72	43	<0.01
Flavored milk	13	18	0.01
Sports drinks	4	7	0.02
Soda	3	4	n/s
**When your child is walking around during the day with something to drink, what are they usually drinking?**			
Tap water	29	41	<0.01
White milk	40	39	n/s
Bottled water	43	37	0.03
Fruit juice	55	31	<0.01
Flavored milk	7	9	n/s
Sports drinks	2	4	0.04
Soda	1	2	n/s

*n/s, not statistically significant*.

*χ^2^-test of association not suitable for tables that include multiple response variables. Analysis of variance was not used since the data are ordinal rather than interval*.

*p < 0.05 is significant.

*χ^2^(4, *n* = 1,203) = 21.2754, *p* < 0.001*.

*ANOVA calculation was not used because the data are ordinal*.

### Policy

Delta Dental of Colorado Foundation provided specific comments and requests in the draft rules ultimately approved in late 2015 by the Colorado State Board of Human Services for all licensed childcare centers in Colorado. The rules included tighter nutrition standards, specifically prohibiting centers from serving sugar-sweetened beverages to children (the rules did not prohibit parents/caregivers from sending their child to a center with a sugar-sweetened beverage). DDCOF encouraged the state board to consider including flavored milks in the list of prohibited sugar-sweetened beverages, and this change was made (flavored milks were not included in the draft released by the state board for public comment). In addition, the rules limited 100% fruit juice to twice per week.

## Discussion

Delta Dental of Colorado Foundation implemented a large, multifaceted public will building program, known as “Cavities Get Around,” between 2014 and 2015. The campaign’s development was informed by the low-income families it was designed to reach in these first 2 years. The program included a range of community interventions, from targeted advertising and public relations to well-trained promotores de salud, community organizers, text messaging, and various health educators who disseminated the key messages to families and communities. The program culminated in the sharing of two key messages: importance and safety of drinking tap water and the impact of drinking fruit juice on children’s baby teeth and oral health. In random surveys of two unique populations living in Colorado in 2014 and 2015, various oral health reported characteristics changed among parents/caregivers, including improved perception of the importance of children drinking tap water, reported increase in children’s consumption of tap water, reported decrease in the perception of fruit juice consumption as healthy for children, and reported decrease in children’s consumption of fruit juice. While we cannot account for the influence of outside secular trends on participants’ reported characteristics, we were encouraged to see an improvement in the perceptions and behaviors targeted with the program. The impact of broad public health campaigns is hard to measure but our results suggest that our public will building program may have had an important impact on Coloradan families. We also were encouraged that 39% of participants statewide and 46% of participants in the Denver metro area who were surveyed in 2015 could recognize our advertising, indicating its penetration in the target communities and that the campaign messages were memorable. The campaign also made inroads with public policy by working within a large coalition of health-focused organizations to successfully advocate for healthier nutrition standards for licensed childcare centers in Colorado.

Other Colorado efforts have primarily targeted reduction of sugar-sweetened beverages, such as soda. Our work was unique from other Colorado efforts in that we focused on raising awareness of the harmful effects of sugar in fruit juice on children’s oral health and on promoting the benefits of tap water. We are not aware of any other efforts with a similar focus on fruit juice and tap water.

Rather disturbing were the statistically significant reported increases (see Tables 7 and 8) in flavored milk consumption among children in the surveyed population, especially 2- to-3-year -old (+16%), children living in the Denver metro area (+12%), and Hispanic children (+14%). We are left to speculate on what may have contributed to reported large increases in children’s flavored milk consumption in the Denver metro area, including the potential that some families reached by the “Cavities Get Around” campaign may have replaced serving their child fruit juice with flavored milks.

**Table 8:** Comparison of results from first and second statewide surveys, assessing family attitudes and behaviors related to drink consumption among Hispanic children.

	April 2014 (%), *N* = 264	November 2015 (%), *N* = 246	*p*-Value*
**Which of the following types of beverages does your child drink? How often do they drink… (% “daily”)?**			
Tap water	34	60	<0.01
Bottled water	60	67	n/s
Fruit juice	68	46	<0.01
Flavored milk	15	29	<0.01
White milk	93	62	<0.01
Sports drinks	3	8	0.01
Soda	1	3	n/s
**How important is it for your child to drink (item) for their health and nutrition? (% “extremely” and “very important”)**			
White milk	95	89	0.01
Tap water	41	52	0.01
Bottled water	68	64	n/s
Fruit juice	73	41	<0.01
Flavored milk	18	25	0.05
Sports drinks	4	10	0.01
Soda	4	6	n/s
**When your child is walking around during the day with something to drink, what are they usually drinking?**			
Tap water	22	34	<0.01
White milk	33	41	n/s
Bottled water	46	51	n/s
Fruit juice	56	27	<0.01
Flavored milk	8	7	n/s
Sports drinks	0	5	<0.01
Soda	1	3	n/s

*n/s, not statistically significant*.

*p < 0.05 is significant.

*χ^2^(4, *n* = 1,203) = 21.2754, *p* < 0.001*.

*ANOVA calculation was not used because the data are ordinal*.

### Limitations

This study has limitations. Though sampling methods used for the first and second household surveys were similar, the respondents were different, making it impossible to know if attitudes and behaviors changed in specific families or individuals. However, random sampling was an effective means for measuring impact of the paid media aspects of the campaign between the baseline and follow-up surveys. In addition, responses given for the household surveys were self-reported and were unverified by observation. Also, we cannot account for the impact of societal trends on the oral health perceptions and behaviors targeted with this work.

## Conclusion

### Lessons Learned

In Phase I, we chose the obvious path for a children’s oral health campaign—tooth brushing—in implementing the public will building initiative in Colorado. Perhaps this was because tooth brushing seemed the highest-impact way to get parents actively involved in preventing caries in their children. This was what parents told us in early focus groups, as well. Overlooked in our Phase I exploratory work was assessing underlying attitudes about baby teeth and the need for a central value proposition: in our case, the importance of baby teeth and the spread of cavities from baby teeth to adult teeth. If the importance of baby teeth was not firmly established, any attempt to change behaviors, social norms, and environments—and build public will for children’s oral health—might fall short. Establishing the importance of baby teeth and a simple way to do so (serve children only water between meals and at bedtime) became the central aim of the Phase II “Cavities Get Around” campaign and it led to strong results. Others involved in children’s oral health campaigns and programs may want to consider building their messaging around the central value proposition of why baby teeth matter.

In addition, as we found in our Phase II research, the greatest knowledge gap was not the need to brush children’s teeth. It was why baby teeth mattered and the adverse impact of fruit juice, consumed between meals and at bedtime, on baby teeth and the oral health benefits of tap water. These knowledge gaps needed to be addressed not just in homes but also in childcare centers, schools, and similar environments influencing children’s oral health. Just focusing on promoting healthy behaviors in the home would have been limiting.

The Flint, Michigan water tragedy also served as a lesson learned. In that case, children in Flint, an economically depressed area, were exposed to lead in the public water system. The Flint crisis publicly surfaced shortly after the 2015 survey concluded. In the wake of Flint, we gathered as much information from local water utilities as possible and used these learnings to modify our messaging to urge families to drink water from whatever source they were most comfortable, though we did continue to emphasize the benefits and safety of fluoridated tap water. Had we not made this change across all facets of the campaign, we believe our overall message might have lost credibility among those we wished to reach.

### Campaign Today

The results from 2014 to 2015 exceeded our expectations. The campaign today remains focused on the same messages related to the importance of baby teeth and children drinking only water between meals and at bedtime but has evolved. For example, in parent workshops at schools, the oral health impacts of unlimited fruit juice consumption continue to be an emphasis, but within a larger conversation about the impacts of *all* sugary drinks on children. In 2016, the campaign replaced the “sugar troll” commercials with a new set of advertisements that more clearly communicated the desired behaviors and expanded the issue to a community frame. Reflecting feedback from another set of focus groups, the commercial used three scenes to reveal how much sugar is in various containers of fruit juice. The scenes—two at home and one at a youth soccer game—show containers that look like fruit juice but are actually full of sugar. The advertisement ends with a young girl reaching for a glass of tap water, with the voice-over saying, “healthy teeth and bodies need water.”

In addition, the campaign is making concerted efforts to affect institutional policy change to create new positive norms and expectations for children’s oral health. In Southwest Denver, partner Westwood Unidos has focused on family education, parent advocacy, school wellness, and training and mentoring of resident leaders and other influencers to spearhead policy change in schools and other community settings. Evidence may suggest that children in schools where sugary drinks are restricted have fewer cavities (17). Public relations tactics are then used to disseminate these institutional policy changes, such that they may be emulated in other parts of the community or state. In 2016, news coverage of our work included a feature in *The New York Times* (18). In 2017, we are developing an activation kit for Colorado communities to use to expand access to drinking water for children in schools and other public spaces.

Meanwhile, since 2015, the *Healthy Beverage Partnership* (HBP), representing Denver metro county health departments, has launched efforts to reduce sugary drink consumption in the region, specifically related to type 2 diabetes, obesity, cardiovascular disease, and tooth decay. HBP uses paid media for its “Hidden Sugar” campaign to raise awareness; works with community organizations to adopt healthier vending, meeting, and concession policies; and supports community coalitions to help promote model policies. The “Cavities Get Around” campaign and HBP share information to allow for advertising efforts to complement each other rather than duplicate efforts. In addition, Delta Dental of Kansas has brought the advertising components of the “Cavities Get Around” campaign to various markets in its state.

### Future

Delta Dental of Colorado Foundation is committed to supporting efforts to raise awareness of the importance of children’s oral health, to the extent that children’s oral health is valued and prioritized in homes, childcare centers, schools, social service programs, and communities. The campaign may expand its grassroots outreach in the immediate years ahead. If the campaign focused only on behavior change and were not also working with residents to change community social norms, expectations, and environments, we speculate that it is unlikely progress could be sustained. “Old habits die hard,” except in cases where these habits have become socially unacceptable. We are working toward a future with better oral health and overall health, when children drink mainly water and have healthy choices at and between mealtimes.

A third statewide survey will be conducted in late 2017, and the data from it will be measured against 2014 and 2015 results to assess progress and identify new and ongoing opportunities for focus. DDCOF will also continually monitor statewide data on rates of caries in children to gauge impact.

## Author Contributions

WH is the lead author and is campaign director for this program. WB is a public health dentist from the University of Colorado and an expert adviser to the campaign. PB is a pediatrician from the University of Colorado and Denver Health and an expert adviser to the campaign. KW owns the firm (HealthCare Research, Inc./Market Perceptions, Inc.) that has provided research and evaluation services to the campaign. He is a trained statistician. JH works at HealthCare Research, Inc., where he has facilitated all focus groups and helped synthesize findings.

## Conflict of Interest Statement

The authors declare that the research was conducted in the absence of any commercial or financial relationships that could be construed as a potential conflict of interest. The reviewer, TT, declared a shared affiliation, though no other collaboration, with two of the authors, WB and PB, to the handling editor, who ensured that the process nevertheless met the standards of a fair and objective review.

## Footnotes

^1^Defined as containing 100% fruit juice.

^2^The “Brush with Me” campaign had been retired and entirely replaced with “Cavities Get Around.” See www.CavitiesGetAround.com (English) and www.LasCariesSePropagan.com (Spanish).
